# Editorial: Women in neuropharmacology: 2021

**DOI:** 10.3389/fphar.2023.1198876

**Published:** 2023-04-12

**Authors:** Divya Vohora, Nidhi Agarwal

**Affiliations:** ^1^ Neurobehavioral Pharmacology Laboratory, Department of Pharmacology, School of Pharmaceutical Education and Research (SPER), Jamia Hamdard, New Delhi, India; ^2^ Centre for Translational and Clinical Research, School of Chemical and Life Sciences (SCLS), Jamia Hamdard, New Delhi, India

**Keywords:** neuropharmacology, neurodegenarative disease, psychiatric diseases, CNS–central nervous system, women in science

## Introduction

Neuropharmacology is a field of study focussing on understanding the actions of drugs affecting the nervous system and encompassing all aspects of research including, *in vitro*, *in vivo* or clinical research on drugs that could potentially treat neurological and psychiatric disorders. Considering the complexity of the nervous system and limited understanding of the etiopathogenesis of most neurological diseases particularly neurodegenerative diseases, there is a critical need to encourage research in this direction. Additionally, there are still unmet needs when it comes to drug therapies for these conditions.

Women in Neuropharmacology 2021 is a part of the inaugural series of article collection for the Frontiers in Pharmacology Research Topics with an aim to acknowledge and promote the contributions of woman scientists in all areas of Neuropharmacology. This is because as per UNESCO fact sheet 2020, there is a significant gender gap for women in science and women researchers represent only 30% of the researchers worldwide (UNESCO Institute for Statistics, 2020). This article collection is a small effort to bridge this gap to highlight the important research findings of woman scientists from all over the world. The collection comprises of 5 original research articles, 3 mini reviews and 1 systematic review from woman authors from 7 countries–Brazil, Denmark, Greece, Japan, New Zealand, UK, USA.

**FIGURE 1:**
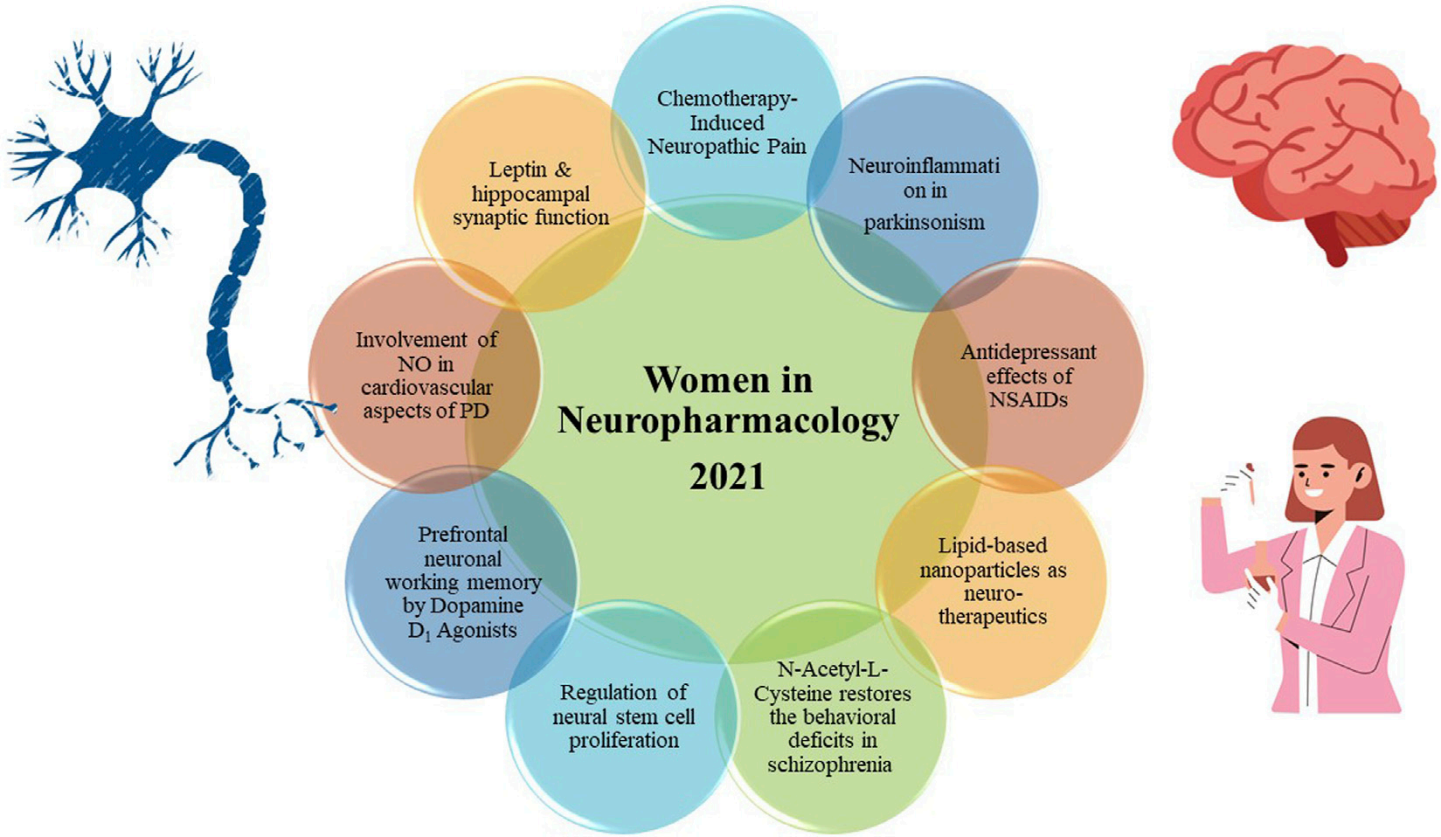
Highlights of articles from Women in Neuropharmacology 2021 Collection.

The Research Topic covers the diverse areas on Neuropharmacology including identifying or reviewing current targets and challenges associated with drug treatments in neurodegenerative diseases such as Parkinson’s disease, Alzheimer’s disease and other neurological diseases such as Schizophrenia, neuropathic pain, depression, learning and memory etc. (Figure 1).

The major highlights from the 9 articles are summarized below:

1. Kappa opioid receptor (KOR) agonists were studied in mice for paclitaxel-mediated neuropathic pain by Kelly Paton from Victoria University of Wellington, New Zealand and coworkers. The major highlight of the study was that the authors evaluated sex differences in view of the fact that women have increased pain sensitivity and respond differently to pain medications and that majority of preclinical studies have been performed in male animals only. The increased antinociceptive effect observed in females was related to a slower elimination in pharmacokinetic studies (Paton et al.). The study indeed complements the recent guidelines by NIH and ARRIVE where researchers must consider sex as a factor while designing, analysis or reporting of research findings, whether preclinical or clinical.
2. Leptin, adipocyte derived hormone, is widely known to have a fundamental role in regulating energy balance. However, over the past several years, leptin role has been reported to be more widespread as leptin receptors are not just restricted to hypothalamus but have been identified in hippocampus and hippocampal synapses. Leptin therefore shows several central actions including precognitive effects. Also, alterations in leptin levels have been linked with several neurodegenerative diseases. Jenni Harvey from University of Dundee, UK highlighted some of these actions and mechanisms thereof through a mini review (Harvey).
3. Yang Yang and coworkers from Penn State College of Medicine, PA, USA (Yang et al.) studied the signalling mechanisms for dopamine D1 receptor agonists at the neuron population level. This is because of the differential effects of D1 agonists on memory exhibiting an inverted-U type response. Their findings suggested that D1-related regulation of memory can be differentially modified by using functionally selective ligands. The authors emphasized that pharmacokinetics and signalling must be considered beyond receptor selectivity.
4. The mini review by Martins-Pinge and coworkers from Universidade Estadual de Londrina- UEL, Londrina, Brazil (Martins-Pinge et al.) presented the role of nitric oxide (NO) in cardiovascular and autonomic dysfunctions observed in Parkinson’s disease. Based on the review, the hypothalamic nuclei were reported to be the target for NO alterations.
5. Maria Tsakiri from National and Kapodistrian University of Athens, Athens, Greece (Tsakiri et al.) presented a mini review on lipid-based advanced RNA-delivery platforms including Liposomes and lipoplexes, solid lipid nanoparticles and lipid nanoparticles, their advantages, disadvantages. Though there were challenges, lipidic nanoparticles for RNA delivery demonstrate potential in the treatment of neurodegenerative disorders, such as Alzheimer’s disease, or brain tumors like glioblastoma.
6. Bay Richter and coworker from Aarhus University, Denmark (Bay-Richter and Wegener) performed a systematic review of preclinical studies of NSAIDs on depressive-like behavior in rodents whereby 36 meeting the inclusion criteria were reviewed. One of the observations was that majority of the studies were conducted in male animals and very few in female animals again highlighting the need to have future studies conducted in both sexes. Selective COX-2 inhibitors in the stress models provided most robust antidepressant effect as compared to the non-selective ones. The mechanisms suggested were related to attenuation of neuroinflammation, HPA Axis dysregulation and alterations of monoamines.
7. Cunha from Universidade Federal de São Paulo, São Paulo, Brazil and coworkers (Cunha et al.) investigated and proved the role of neuroinflammation in repeated low-dose reserpine-induced model of Parkinson’s disease in Wistar rats using immunostaining for astrocytes and microglia markers and cytokines expression. The neuroinflammation was observed in regions involved in the pathophysiology of PD and reversed 20 days later.
8. Lopes-Rocha and co-workers from Universidade Federal do ABC, Brazil (Lopes-Rocha et al.) reported the antipsychotic effects of antioxidant N-acetyl-L-cysteine (NAC) in metilazoximetanol acetate (MAM)-induced neurodevelopmental model of schizophrenia in Wistar rats through a mechanism that involves nitric oxide. Based on the findings, the authors suggested oxidative stress to be a potential target for schizophrenia.
9. Recent research has shed new light on the regulation of NSC proliferation and survival by Protein Arginine Methyl Transferase-1 (PRMT-1). PRMT-1 is necessary for NSC proliferation, and loss of PRMT-1 activity leads to decreased NSC proliferation and survival. The research has important implications for the development of potential therapies for neurological disorders. The research conducted by a team of scientists led by Misuzu Hashimoto at the Gifu University of Japan (Hashimoto et al.) examined NSCs derived from Nestin-Cre mediated *Prmt1*-deficient mouse embryo both *in vitro* and *in vivo*. The study concluded that PRMT1 plays a cell-autonomous role in the survival and proliferation of embryonic NSCs.

## Concluding remarks

Research in neuropharmacology is essential for our understanding of the intricate nature of nervous system and developing new treatments for neurological and psychiatric diseases. This article collection highlighted the novel CNS actions of some of the key targets such as leptin, PRMT-1, KOR agonists; the new indications of existing treatments such as NSAIDs in depression, NAC in psychosis, D1 receptor agonists in memory; role of neuroinflammation and NO in animal model and addressing the complications of Parkinson’s disease respectively; and lipid-based advanced RNA-delivery platforms for neurodegenerative diseases. Further, one of the articles reported that there might be differential effect of drugs when studied in male and female animal models of disease. So, as far as we encourage to bridge gender gap in science, we must also bridge this gap in experimental research and consider sex as an important biological variable.
